# Approaches to Laparoscopic Training in Veterinary Medicine: A Review of Personalized Simulators

**DOI:** 10.3390/ani13243781

**Published:** 2023-12-08

**Authors:** Cosmina Andreea Dejescu, Lucia V. Bel, Iulia Melega, Stefana Maria Cristina Muresan, Liviu Ioan Oana

**Affiliations:** Department of Surgery, Anesthesiology and Intensive Care, Faculty of Veterinary Medicine Cluj-Napoca, University of Agricultural Sciences and Veterinary Medicine Cluj-Napoca, 400372 Cluj-Napoca, Romania; cosmina.dejescu@usamvcluj.ro (C.A.D.); iulia.melega@usamvcluj.ro (I.M.); stefana.muresan@usamvcluj.ro (S.M.C.M.); oanaliviu2008@yahoo.com (L.I.O.)

**Keywords:** laparoscopy, domestic animals, training, veterinary simulators, veterinary surgery, minimally invasive surgery

## Abstract

**Simple Summary:**

The field of veterinary minimally invasive surgery has grown, but there is a lack of easily accessible training tools for practitioners worldwide. While borrowing human medicine simulators helps with basic skills, specific veterinary procedures require specialized training. Some simulators are being developed, validated, and found to be effective for training vets both in basic skills and advanced techniques. Nevertheless, these simulators are of interest mostly for dogs and horses, and their number is small. This study emphasizes the need for more advanced simulators to improve surgical techniques for various animal species.

**Abstract:**

Veterinary minimally invasive surgery (MIS) has experienced notable growth in recent years, yet the availability of specialized training tools remains limited and not readily accessible to practitioners worldwide. While borrowing simulators from human medicine practices suffices for acquiring fundamental laparoscopic skills, it proves inadequate when addressing procedure-specific nuances. Veterinary professionals are now taking steps to create simulators tailored to their patients, although the validation process can be time-consuming. Consequently, the availability of advanced laparoscopic simulators for veterinary training remains scarce. The present study aims to highlight custom-made simulators. A comprehensive search across five databases was conducted to uncover the simulators documented from 2010 to 2022. A total of five simulators emerged from this search, with four grounded in a canine model and only one in an equine model. These models underwent validation and were found to be effective in training surgeons for their designated tasks. The findings underscore a limited array of simulators, predominantly catering to two species (horses and dogs). Considering these findings, it is evident that further research is imperative to create laparoscopic simulators capable of facilitating advanced veterinary training. This would enable the continued evolution of surgical techniques across diverse species, including ruminants, small mammals, and non-mammalian animals.

## 1. Introduction

Veterinary practitioners encounter a diverse array of species under their care, ranging from tiny songbirds to massive elephants. However, size is not the sole contrasting factor among these patients. Significantly distinct anatomical and physiological characteristics exist between herbivorous and carnivorous mammals, birds, reptiles, and other categorizations. Recognizing this diversity underscores the understanding that uniform medical approaches cannot be universally applied to all patients. Therefore, specialized training becomes imperative to delivering optimal care, particularly in the domains of surgery and minimally invasive procedures within the field of veterinary medicine.

Minimally invasive surgery (MIS) is an area of medicine that first was developed in human medicine and over the years was borrowed and translated into veterinary medicine. The concepts of MIS are based on reaching the inside of natural cavities such as the thorax, abdomen, joints, and urinary bladder through small incisions or through natural orifices (natural orifice transluminal endoscopic surgery—NOTES), having better visualization of the surgical field by magnifying the images, a decrease in blood loss and post-operative pain, leading to a faster recovery time. The difficulties of performing MIS procedures are represented by using different and more expensive equipment, the “unnatural” position in which the surgeons have to stay during surgery, and some pathologies of the patients that make MIS a contraindication. The difficulties encountered during MIS include a magnified tremor and a limited tactile sensitivity (haptic feedback) due to the use of long instruments, limited movement of the instruments in the port (fulcrum effect), the loss of depth perception as the vision shifts from binocular to monocular, and the loss of the “bird’s eyeview” as the field of view is going to be reduced and every instrument outside the field of view can become a liability. Under these conditions, the training for minimally invasive surgery is different from the one for open surgery and it is based on two components: basic skills and advanced training.

Laparoscopic basic skills can be achieved and practiced with a set of exercises that are confirmed to improve ambidexterity, hand–eye and hand–hand coordination, instrument targeting accuracy, and the recognition of cues for the sense of depth. These exercises were first used by the Fundamentals of Laparoscopic Surgery (FLS) in human medicine and then transferred into veterinary surgery with the help of the Veterinary Assessment of Laparoscopic Skills Program (VALS). The exercises consist of peg transferring, pattern cutting, ligature loop placement, and intra and extracorporeal suturing. All of these can be practiced using box trainers, ranging from a homemade one to a complex human trainer, as no differences were shown to be relevant between a low-cost and a high-cost trainer [1], and veterinary students can achieve proficiency on an FLS simulator [2]. Another study concluded that basic laparoscopic skills can be obtained through a variety of simulators [3].

Conversely, achieving proficiency in advanced veterinary minimally invasive surgery presents a greater challenge due to the significant variation in patients and surgical methodologies. Consequently, relying solely on methods such as box trainers or learning from human trainers falls short of meeting the requirements of veterinary surgeons. The demand for more sophisticated training tools arises. High-fidelity simulators encompass a range of options, including working with live animals, utilizing cadavers, employing simulated digital imagery through virtual or augmented reality (VR/AR), and employing task-specific, custom-built physical simulators. Each of these approaches carries its own distinct advantages and drawbacks [4,5].

The need for costumed simulators was noticed by many clinicians. Fugazzi and colab. [6] evaluated a neoprene model to replace the diaphragmatic muscle in a simulation of closing diaphragmatic defects, as using cadaveric tissue led to several deficiencies. They stated that the use of a single fabric-sided neoprene model has the potential to replace biological tissue in the simulation of diaphragmatic herniorrhaphy [6].

The first simulator made specifically for veterinary MIS was reported in 2010, using a canine model [7,8]. Since then, a small number of veterinary simulators have been developed and validated. The validation process is of utmost importance after building a simulator, as it assesses the resemblance and efficiency of the simulator. Various validation frameworks have been designed, with concurrent validation being the one that is most decisive [5,9].

A study from 2018 questioned the experience of veterinary surgeon residents enrolled in the American College of Veterinary Surgeons’ (ACVS’s) small animal residency program with MIS and simulator training. Less than half of the respondents had access to a simulator. Furthermore, the training time was limited because of the busy schedule. The residents who had access to simulators felt more prepared to perform MIS, in contrast to their colleagues who did not have this kind of training available. The authors of the article recommended that simulation training should be made accessible to residents and that the schedule should be organized so time can be set aside for MIS training. Moreover, further research should be conducted regarding the types of simulators and training curricula [10]. The consensus among surgeons appears to be that an optimal simulator should be easily accessible, and proficient in imparting both fundamental and advanced skills, all while remaining cost-effective in terms of training expenses (Table 1).

This narrative-scoping review article seeks to identify and outline the laparoscopic simulators that have been specifically created for the purpose of advanced training in veterinary minimally invasive surgery (MIS). Additionally, the study aims to assess the feasibility of employing these simulators on a larger scale within the veterinary field.

## 2. Materials and Methods

The focus of this study was on manuscripts in which specific veterinary laparoscopic simulators were described. The descriptions, origins, costs of production/price of acquisition, level of training (basic skills/advanced skills), and validity were studied. Parts of the article text were subjected to proofreading and rephrasing using Artificial Intelligence Software (ChatGPT-3.5) before being submitted. The reference list was managed and organized using Mendeley Reference Manager.

### Search Strategy

PubMed, Scopus, the Web of Science Core Collection, ScienceDirect, and the Wiley Online Library were searched for papers on the development and validation of veterinary minimally invasive simulators using the search terms and keywords ”laparoscopic/laparoscopy veterinary simulators” and “minimally invasive surgery abdominal veterinary simulators”. Subject refinement to “Veterinary Medicine” was deemed necessary for the Wiley Online Library and Science Direct database searches. The Google Scholar database was excluded due to its high number of results and lack of clear refining tools. All databases were searched between January and March 2023. Data coverage was represented from January 2010 to December 2022. Language restriction was not established, but an English abstract had to exist in order to find the articles. All results were collected in a single database and duplicate entries were removed. A “snowball” search was used to complete the database. Furthermore, commercially available simulators were included. All animal species were included. Articles’ titles, abstracts, and full-texts were searched. Several articles were scanned for the use of a specific simulator in the protocol study and further research was performed.

The exclusion criteria were as follows: the use of non-specific veterinary simulators (human simulators, box trainers, simulators based on more than one animal), vivo/ex vivo animal models, cadaveric models, the full text was unavailable or articles were not relevant for the research, and the publication date was before 2010. Inclusion criteria consisted of articles relevant to the search where at least an English abstract was provided.

## 3. Results

The search strategy yielded a total of 198 articles (PubMed—48, Scopus—22, Web of Science Core Collection—41, Science Direct—8, Wiley Online Library—79). A total of 50 articles were removed due to their being duplicates. After an initial scan of the title and abstract, 107 articles were excluded for not meeting the predefined criteria. A full-text was not available for 5 articles, and 8 more articles were excluded after a full-text analysis.

The Google Scholar database search generated 3410 results for “laparoscopic veterinary simulator,” 3370 for “laparoscopy veterinary simulator,” and 15,500 results for “minimally invasive surgery abdominal veterinary simulator.” Given the vast number of results and the lack of clear methods for refining the search, we opted to exclude this database from our search strategy.

The findings from the 28 articles that were included indicated a very small number of simulators specifically built for veterinary laparoscopy, with the focus being on the canine model (*n* = 4). Large animal simulators were poorly represented (*n* = 1), and we could not find any simulators for small mammals, birds, or reptiles. The simulators were used both for basic skills training (for example, peg transfer), exploring the abdominal cavity, and for specific surgical techniques such as an ovariectomy or gastropexy in veterinary patients of different species (Table 2).

### 3.1. Equine Laparoscopy Simulators

#### Standing Equine Laparoscopic Ovariectomy Simulator (SELO)

Only one simulator regarding equines was found in the researched literature. It was developed by Elarbi M. and collab. in 2018. The model is called the SELO simulation model, and its purpose is to train veterinary surgeons in performing laparoscopic ovariectomies on standing horses. The simulator was based on the external and internal measurements of healthy horses and mules, patients undergoing laparoscopic ovariectomies, and the genital tracts harvested from euthanized mares. Low-cost and commercially available materials were used for the construction of the simulator. Therefore, silicone balls, balloons, liquid latex, toilet paper, latex tubing, and hair gel were used for the organs. The abdominal wall consisted of a yoga mat as the outside layer and a table cover as the inside [11].

Other components were represented by a 30° laparoscope, a video monitor, a light source, a webcam, and a laptop. To complete the simulated surgical procedure, laparoscopic instruments such as scissors and grasping forceps were used. A marker pen replaced the injection needle for local anesthetic administration [11].

The simulator was validated by asking fifteen students and four equine surgeons with different experiences to use the simulator and therefore help with the assessment of the face, construct, and concurrent validity [11].

It was concluded that the SELO simulation model is useful for training surgeons and vet students on laparoscopic ovariectomies for standing horses [11].

### 3.2. Canine Laparoscopy Simulators

A high number of veterinary patients are represented by dogs; therefore, most of the simulators found during our research were based on a canine model.

#### 3.2.1. The CLS (Canine Laparoscopic Simulator)

The CLS was developed by a team of surgeons from Spain in 2014 using the computed tomography scans of three beagle dogs during pneumoperitoneum at an intraabdominal pressure of 12 mmHg. The simulator consists of a transparent box made from methacrylate (40 cm × 20 cm × 15 cm), with an oval-shaped base and a doom-shaped ceiling as the workspace that is divided into the thoracic and abdominal cavities [12]. The background is represented by an anatomical drawing of the abdominal and thoracic viscera. It has nine access ports for the laparoscopic instruments and two cranial and caudal ports for endoscopic access. The simulator can be used both with an integrated camera and light or a telescope. It allows for the practice of basic laparoscopic skills, NOTES, and single-port MIS procedures [13].

The evaluation process consisted of having two groups of veterinarians (seven experts and twenty-three novices) complete four tasks on the CLS. After the exercises were completed, the surgeons answered a survey regarding the experience. It was concluded that the CLS was a good training instrument for veterinarian surgeons [13]. In a further study, the authors found the CLS to be suitable for training and teaching after assessing the face and construct validity using a group of 12 experts and 30 novices [14].

The simulator is available for online purchase in Spain only. It can be found on the Jesus Uson Minimally Invasive Centre website under the name SIMULVET^®^ [12]. Another version of the simulator can be found on the same website under the name SIMULAP^®^ [15]. Different synthetic organs can be bought from the same site.

#### 3.2.2. The CALMA Veterinary Lap-Trainer Composite Simulator (CLVTS)

The need for more advanced training in minimally invasive canine surgery led to the development of the CLVTS for total laparoscopic gastropexy (TLG) training. The box was created with the help of two Great Dane dogs as models. A plaster bandage was used to form a negative mold in exactly the same shape as the dogs during the insufflation of CO_2_. The positive mold was obtained after layering the plaster mold with fiberglass and epoxy resin on the inside. Therefore, it resulted in a box that simulated the abdomen all the way to the mid-thoracic region of a giant breed dog with the dimensions being 46 cm long and 30 cm wide at the base. The height of the box varies along different aspects of the “body”, being 27 cm in the “sternal region” and 14.6 cm in the “pelvic region”. The total workspace was approximately 15,000 cm^3^. The design of the mold included five holes in the ventral part and two additional inferior ports. In the area corresponding with the gastropexy site, a silicone pad was placed. The simulator has LED lighting and an internal digital camera. It used a pig stomach for performing the gastropexy [16].

The validation of the CLVTS was performed by two groups of veterinary surgeons with different levels of surgical experience (six advanced-experienced and ten non-experienced). All participants had to execute four exercises to complete the task (TLG): anchoring sutures, cutting exercises, and suturing exercises. The conclusion of the study was that the CLVTS was accepted as a realistic and useful tool for teaching and training intracorporeal suturing for TLG [16]. In a more recent paper regarding the face, content, construct, and concurrent validity assessed by four experts and ten non-experts, the authors showed that the CLVTS can be successfully used also for basic skills training in MIS [17]. The Objective Structured Assessments of Technical Skills (OSATS) and Hands Movement Assessment System (HMAS) were used to evaluate the surgical skills. The CLVTS was demonstrated to be useful for gaining surgical skills and transferring them into the operating room [18].

#### 3.2.3. Canine Simulated Laparoscopic Ovariectomy Model (SLO)

The model proposed by a team from Washington State University used a commercially available canine simulator (Canine MESI torso abdomen model, Sawbones, Vashon, WA, USA) and created the genital apparatus from plaster using clay. Silicone was used for the molding of the ovaries and uterus. The silicone was mixed with dye to obtain the different colors of the organs, ligaments, and adipose tissue. Three marks were placed in key sites: the suspensory ligament, proper ligament, and ovarian pedicle. These marks were used for dissection during the simulated ovariectomy. The model was placed inside the simulator in a topographic position. The spleen model that was included in the initial kit of the abdomen model was situated just ventral to the entry site, and a pressure-sensitive paper was fixed above it [19].

The instruments used were standard and consisted of cannulas, a 10-mm rigid 30° telescope, a blunt probe, curved forceps, and a 10-mm vessel-sealing device. Images were recorded with the use of a high-definition webcam set in a fixed position [19].

Three groups of veterinary students and surgeons with different levels of experience were asked to participate in the process of validation. The SLO construct’s validity was tested by having the participants complete the laparoscopic ovariectomy. The concurrent validity was established using the basic skills tasks (pegboard transfer, pattern cutting, ligature loop placement). A questionnaire was completed to evaluate the face validity. The study concluded that the SLO has good construct and concurrent validity, but its face validity could be improved [19].

#### 3.2.4. Mayo Endoscopy Simulated Images (MESI) Canine Abdominal Model

This simulator was used in many studies regarding the training of veterinary minimally invasive surgery [8]. It is cited as validated for being used by veterinary surgeons for training in both basic and advanced skills [5,20]. Some researchers used the MESI model as a starting point for training in procedures such as ovariectomy [19]. The box has a rounded shape similar to the canine abdomen during pneumoperitoneum and is made from hard black plastic [21]. It has six premade holes placed strategically to have access to the abdominal organs during the simulated procedures. Inside the box, several organs such as the liver, spleen, and intestinal loops are anatomically placed [19]. No information regarding the construction or availability of the MESI model at present was found by the authors.

## 4. Discussion

Veterinary surgeons use human-designed trainers to exercise their basic skills, but the variety from an anatomical and patient-size point of view makes training for specific tasks difficult and not realistic. The findings of this review revealed a very small number of specifically built physical simulators for veterinary minimally invasive surgery. The most represented species was dogs, with four out of five simulators being canine models (80%). The other simulator was used for equines. This finding is similar to the results of another paper, in which 61.6% of the veterinary simulators were for small animals (73.3% canine), 13.7% for farm animals, 12.3% equines, and the rest were applicable to all species [22]. No models for farm animals or other small animals were found during our research. It is obvious that although the interest in minimally invasive surgery is rising, as two more canine simulators were developed since 2020 when only the MESI and CLS models were described [5], most of the species veterinarians work with are underrepresented or not represented at all in MIS training. Spaying female rabbits is recommended, as they are at high risk for developing uterine adenocarcinoma. Keeping in mind the sensitive nature of the rabbit abdomen, a laparoscopic approach would be beneficial for patients, but most complications of this procedure are due to the absence of proper training [23]. Although simulators for practicing different medical maneuvers are available for large ruminants [24], MIS simulators of this kind were not found during our research. Multiple papers revealed the need for practicing minimally invasive abdominal surgeries such as abomasal cannulation [25], abomasopexy [26], or genitourinary procedures [27,28] in these patients; therefore, a simulator on which surgeons can safely train is necessary.

Although the costs of production are not specified in the researched papers, a tendency to lower the costs of simulators was observed, as cheaper materials and technologies were used for constructing the models. Silicone was considered to be more feasible as it allowed itself to be poured into different shapes. Its properties made it the most suitable to mimic the feeling of a real organ and provide the haptic feedback that is so valuable during surgery but diminished during MIS [29]. The budget-friendly use of silicone involves inserting it into a rigid mold of the shape needed. This is the way that the SELO and SLO organ models were created. The CLVTS box was built by applying resin to the mold. Another way to obtain abdominal viscera or walls based on silicone or resin is through 3D printing. The disadvantages of this option are the higher initial investment in the acquisition of a printer and the necessity of a trained operator [30]. A study showed no difference between the results of the trainees during basic laparoscopic skills training on a low-cost trainer and on a more expensive trainer box [1], but the impact using cheaper simulators for advanced training has over the results is yet to be determined.

All the simulators were validated using the classic framework for assessing the content, construct, concurrent, and face validity [9]. The evaluation was performed in one or two stages. The results were good for most, except for the face validity of the SLO model, where it was stated that it could be improved [19]. The importance of the validation of the newly built simulators is to assess whether they can be used efficiently for training, and not only if the trainee is improving their skills on the simulator, but if the skills are transferable to the surgical unit during live surgeries. The subjective and objective analyses of the simulator help to gather information about how the surgeon reacts to and interacts with the trainer, but also if the simulator is realistic and the results are comparable to the ones from already validated simulators. A study showed well-transferred skills to the surgical theatre after training on a human simulator inside of which a canine female genital tract made of silicone was placed [31]. The outcome of that study was measured using the Objective Structured Clinical Examination (OSCE) format and the OSATS. These two tools provide means to objectively analyze and score surgical performance in MIS [32,33,34]. The HMAS is another method that can be used to assess the hand motion of the surgeon during training through the instrument called the Iglove. In a preliminary study, the HMAS showed results that correlate with what is considered to be the gold-standard simulator, the LapSim (Surgical Science^®^) [35].

An essential aspect of developing surgical skills also hinges on the trainee’s mindset. The efficacy of a simulator lies in its simplicity with respect to the surgical procedure and its user-friendliness. This ensures that trainees do not become discouraged and abandon their training due to frustrations arising from simulator malfunctions [4].

Most of the equipment used with the described simulators is standard for a variety of laparoscopic surgeries and includes forceps, rigid telescopes, scissors, a needle-holder, etc. A fixed webcam connected to a monitor/computer/laptop can also allow for a lowering of the costs of the training. The light can be provided by using ambient light in open box trainers or boxes with transparent walls (CLS), or an additional light source such as an LED lamp can be used [21].

Although a complex discussion regarding the terminology of “fidelity” has taken place [36], in general, the simulators for minimally invasive surgery are divided into low-fidelity simulators that can be used for basic laparoscopic skills training and high-fidelity simulators used for advanced training. The latest group consists of physical simulators, such as the ones described earlier in this article, live animals, cadavers, real organs obtained from dead animals, and VR and AR simulators. The last group of simulators provides realistic images of the abdomen, instant feedback, and more accurate evaluations of motion, but lacks haptic feedback. Steps are made to incorporate more tactile responses with the new generations, such as the LapSim Haptic System (Surgical Science^®^). Despite the effectiveness of these simulators, they remain cost-prohibitive and will not be spreading soon across training [37]. In a survey regarding arthroscopy, it was found that large animal veterinarians have more access to cadaver training than their small animal correspondents, and the lack of time, supervision, and resources were listed as the main impediments. The other categories of trainers were not used by the majority of the surgeons, with the lack of availability being primarily cited [38].

The use of live animals, cadavers, and animal organs has the advantage of being the most high-fidelity simulator, allowing the trainees to simulate the procedure from beginning to end without missing any steps. From another perspective, live animals or cadavers can be considered the “gold-standard” type of simulation when assessing concurrent validity [9]. On the other hand, the major disadvantages are the need for ethical approval and the small number of repetitions that can be performed [24]. Also, this kind of simulation is not always available, but the process of plastination can help preserve more the specimens for later use [24]. The physical simulators remain the most accessible and reliable. Basic box trainers can be used by both human and veterinary surgeons for basic laparoscopic skills training, but the necessity of advanced simulators is starting to grow as MIS is becoming more and more used in veterinary medicine for different species. Gastropexy is one procedure that a resident of the American Veterinary Surgery College is not allowed to perform on a live patient in some institutions until they have completed and passed the simulation test [39]. This situation makes the CLVTS an important tool in surgical training, but the use of a pig stomach makes it difficult to train in every moment you have time available. One of the most common complications during laparoscopic spaying is puncturing the spleen during the trocar placement [40]. A retrospective study from 2014 revealed that the learning curve for achieving proficiency in the laparoscopic canine ovariectomy procedure is around 80 repetitions of the procedure [41]. This finding makes the use of a specific canine simulator built like SLO needed in order to gain experience and be able to practice in a safe environment. Another issue regarding the use of a universal simulator is that the skills acquired in a certain plane do not transfer when switching planes [42]. This is more important in large animals such as ruminants or equines, where a higher number of procedures are performed on standing patients. Therefore, it is recommended to train both in a vertical position as well as in a horizontal position, but the SELO simulator was the only simulator found to allow for training in a vertical plane.

Alternative approaches to acquiring new skills in minimally invasive surgery (MIS), including video gaming, have been proposed. A 2016 systematic review failed to establish a robust correlation between video games and surgical performance. Nonetheless, a potential shift in the training protocol might reveal contrasting outcomes [43]. This was exemplified in a 2017 study by Sammut et al., where they concluded that prior video gaming experience enhanced baseline laparoscopic performance [44].

This article is not meant to be an exhaustive review of MIS simulators and training, but rather to emphasize the lack of and need for more specifically built trainers for veterinary medicine. Additional research is necessary to assess the reproducibility of the described simulators and how they can be implemented successfully in training programs around the world. Another limitation of this research is the lack of data regarding the costs of developing the simulators, or if they are available for purchase. The MESI model was not found to be purchasable online, while the CLS can be bought only in Spain.

## 5. Conclusions

We concluded that despite the growing interest in performing minimally invasive surgery and training, very few simulators for specific veterinary purposes are available. Clinicians around the world make attempts to improve these training conditions by developing low-cost, high-fidelity simulators. The simulators that are not available for purchase are described in detail, making them easy to replicate.

A focus is seen on small animals such as dogs, but smaller mammals like rabbits and guinea pigs are completely overlooked. Large animal laparoscopies are different since the approach to the patient is in a standing position, making specially built simulators a necessity to improve surgical abilities. The “One size fits all” strategy was never applicable to veterinary medicine and it is even more striking when trying to train for a laparoscopy.

Evaluating the validity of simulators is of extreme importance, but may be a difficult and long process as an ideal number of veterinary surgeons that can provide meaningful feedback cannot be easily reached.

Further studies are needed in order to develop, validate, and implement veterinary simulators. The future focus is to simulate advanced surgical procedures in physical simulators that resemble the patient while keeping a low cost of training. Also, laparoscopic training needs to be affordable for every veterinarian who wishes to train, not just for surgeons working in specialized institutions.

## Figures and Tables

**Table 1:** Properties of an ideal laparoscopic simulator.

Laparoscopic Simulators					
Realistic	Accessible	Basic skills	Advanced training	User-friendly	Low-cost

**Table 2:** Veterinary Simulators for laparoscopy. Species, year of published paper, and procedures available for training.

Simulator	Species	Year of Simulator Development	Procedure
Standing equine laparoscopic ovariectomy simulator (SELO)	Equine	2018	Ovariectomy
The CLS (Canine Laparoscopic Simulator)	Canine	2014	Endoscopy, NOTES, Laparoscopic procedures
The CALMA Veterinary Lap-trainer Composite Simulator (CLVTS)	Canine	2022	Gastropexy
Canine Simulated Laparoscopic Ovariectomy Model (SLO)	Canine	2019	Ovariectomy
Mayo Endoscopy Simulated Images (MESI) canine abdominal model	Canine	Unknown *	Multiple procedures

* Before 2010.

## Data Availability

Not applicable.
